# Colds

**Published:** 1883-11

**Authors:** 


### COLDS.

THE most prevalent of all ailments are colds, affecting various parts of the body; "they have all seasons for their own," and are the beginnings of more diseases than all other causes combined.

It becomes, then, a matter of the first importance to know how to avoid colds, and how to get rid of them speedily when they attack us.

The most frequent cause of colds is wet feet, or feet that remain for a long time damp and chilled from wearing too thin stockings and shoes. It is probable that half of the diseases peculiar to women are induced in that way. It is not the wetting of the feet that gives cold, but the gradual evaporation of the moisture, which carries off the natural warmth of the body, thus causing the blood to be chilled. The effect of chill is to close the pores of the skin, so that the waste particles of matter cannot escape from the body in this direction, but are thrown back and thus poison the blood.

When it is not possible to take off the shoes and stockings, and dry and warm the feet promptly after getting them wet, it is better to let them remain wet until we can attend to them properly. To dry them in the sun or before a fire, without taking off shoes and stockings and substituting dry ones, is extremely hazardous. There is but little danger of taking cold after wetting the feet if we walk rapidly enough to keep up the natural temperature of the body, and keep on walking until we reach some place where we can take off shoes and stockings and thoroughly dry them or change them for dry ones. A cold is less likely to result from a thorough drenching of the whole body than from wetting the feet alone.

People seldom take cold when they are exposed to sudden lowering of the temperature of the air while they are out of doors; they may have their hands and feet frost-bitten, and become almost unconscious from freezing, and yet escape the dangers of an ordinary cold.

A cold in the head may frequently be cut short if treated at once, by snuffing up the nose the fumes of spirits of camphor, ammonia or bay rum. This remedy must be applied every few minutes to be effective. In the meantime the patient should remain in a warm room, and avoid draughts of air. If the cold is not cured in twenty-four hours, it will continue ten or twelve days in spite of treatment, or if neglected its effects may last a lifetime. Colds result in serious and fatal diseases *only* when neglected. We have so often given directions for treating neglected colds that it is hardly worth while to repeat them here. Remain in the house, and if necessary in bed, until the cold has disappeared, and then venture out cautiously at first, as the system is sensitive to fresh attacks for several days after recovery.

